# Fish oil supplementation in obese rats ameliorates metabolic syndrome response

**DOI:** 10.1590/1414-431X2024e13172

**Published:** 2024-05-20

**Authors:** D.M.B. Freitas, B.A.C. Oliveira, L.D.V. Henschel, M.H.A.P.C. Oliveira, M. Zazula, E. Horlem, D.F.S. Rodriguez, S.R.S. Carvalhal, F. Iagher, R. Fernandez, K. Naliwaiko, L.C. Fernandes

**Affiliations:** 1 Departamento de Fisiologia, Setor de Ciências Biológicas, Universidade Federal do Paraná, Curitiba, PR, Brasil; 2 Departamento de Biologia Celular, Setor de Ciências Biológicas, Universidade Federal do Paraná, Curitiba, PR, Brasil

**Keywords:** Obesity, Metabolic syndrome, Innate immune system, Adiposity, Metabolism

## Abstract

Accumulation of visceral adipose tissue is associated with metabolic syndrome (MS), insulin resistance, and dyslipidemia. Here we examined several morphometric and biochemical parameters linked to MS in a rodent litter size reduction model, and how a 30-day fish oil (FO) supplementation affected these parameters. On day 3 post-birth, pups were divided into groups of ten or three. On day 22, rats were split into control (C) and small litter (SL) until 60 days old. Then, after metabolic disturbance and obesity were confirmed, FO supplementation started for 30 days and the new groups were named control (C), FO supplemented (FO), obese (Ob), and obese FO supplemented (ObFO). Comparison was performed by Student *t*-test or 2-way ANOVA followed by Tukey *post hoc* test. At the end of the 60-day period, SL rats were hyperphagic, obese, hypoinsulinemic, normoglycemic, and had high visceral fat depot and high interleukin (IL)-6 plasma concentration. Obese rats at 90 days of age were fatter, hyperphagic, hyperglycemic, hypertriacylgliceromic, hipoinsulinemic, with low innate immune response. IL-6 production *ex vivo* was higher, but in plasma it was not different from the control group. FO supplementation brought all biochemical changes to normal values, normalized food intake, and reduced body weight and fat mass in obese rats. The innate immune response was improved but still not as efficient as in lean animals. Our results suggested that as soon MS appears, FO supplementation must be used to ameliorate the morpho- and biochemical effects caused by MS and improve the innate immune response.

## Introduction

Obesity is a multifactorial chronic disease resulting from the combination of excessive energy intake from diet and decreased energy expenditure (1). It is characterized by an accumulation of adipose tissue underneath the skin and around organs. The hypertrophy of adipocytes leads to dyslipidemia, hypertension, insulin resistance, and low-grade inflammation, which are the main components of metabolic syndrome (MS). Furthermore, obese people may develop diabetes, thus increasing the risk of atherosclerosis, kidney disease, stroke, and major ischemic heart failure (2).

Appropriate animal models, particularly those with dietary intervention, have been used to investigate early obesity and its effects in human adult life (3). An interesting obesity model whose diet is not changed is the rodent litter size reduction in early life (4). In short, at 3-4 days after birth, the litter is reduced to 3 pups per dam, but the control litter is kept with 10 or more pups per dam (5). As a result, the offspring has more access to the nipples since there is no competition for suckling, thus resulting in early postnatal overfeeding, which will lead to obesity and its metabolic disorders at adult stage.

Few studies in humans have investigated associations between consumption of fish and metabolic syndrome. A systematic review by Albracht-Schulte et al. (6) suggested that fish oil (FO) consumption has a protective role in MS prevention, particularly sex-related MS, where men present more benefit than woman. Baik et al. (7), in a Korean follow-up study, investigated the effect of FO and n-3 polyunsaturated fatty acids (n-3 PUFA) consumption on the incidence of MS. They reported that the increase of n-3 PUFA was associated with lower risk of MS, but no significant associations were observed among women. A cross-sectional study from Finland by Kouki et al. (8) investigated the association between consumption of fish and MS development. They reported an inverse association among men, where those with high fish consumption presented lower risk of MS. Moreover, animal studies have achieved solid evidence (9). All these findings indicate that FO consumption may be beneficial in reducing MS by modulation of lipid metabolism and inflammation.

The first evidence of beneficial effects of FO fatty acids on cardiovascular disease was reported by the Danish physicians Bang et al. (10). Since then, FO (rich in eicosapentaenoic acid-EPA and docosahexaenoic acid-DHA) is sought after for health benefits. Long n-3 PUFA may be beneficial in the treatment of conditions with an inflammatory component, such as MS (11). The use of n-3 PUFA is also considered a valuable clinical tool for the treatment of hypertriacylglycerolemia (TAG) (12). Indeed, a reduction of 56% in TAG was seen in rats that received FO (13).

In the present work, we aimed to examine several murimetric and biochemical parameters linked to MS in rats from a litter reduction model at 60 days of age, and how a 30-day FO supplementation affected these parameters. We hypothesized that FO supplementation ameliorates the symptoms of MS.

## Material and Methods

Unless otherwise indicated, all chemicals were purchased from Sigma Chemical Co. (USA). Assay kits for measurements of glucose and triacylglycerol from plasma were purchased from Laborclin (Brazil). Insulin and IL-6 and IL-10 kits were purchased from Invitrogen (USA).

### Animals

All animal protocols were approved by the Ethics Committee for Experimental Animals from the Federal University of Paraná (CEUA number 1274/2019). Animals were housed under a 12-h light/dark cycle at 23±1°C with free access to water and chow. All animals received a regular chow diet (230 g/kg protein, 660 g/kg carbohydrates, 40 g/kg fat, 60 g/kg fiber, and 10 g/kg vitamins and minerals; Nuvital, Brazil).

Food and body weight were monitored every two days from weaning until the age of 90 days.

### Induction of obesity by metabolic programming

Each 90-day-old dam had ten pups. On day three post-birth, one group was kept with three pups per dam, resulting in early overfeeding during lactation. The control group was kept with 10 pups per dam. The pups were weaned at 21 days of age, and then two experimental groups were formed: control (C) and small litter (SL), being maintained as such until the age of 60 days. Then, FO supplementation began and lasted for 30 days, after which four groups were set: control (C), obese (Ob), control fish oil supplemented (FO), and obese fish oil supplemented (ObFO). The rats were given 1 g/kg of body weight per day of FO, which was provided as a single daily bolus through a micropipette. The FO used was a mixed marine triacylglycerol preparation containing 180 g eicosapentaenoic acid and 120 g docosahexaenoic acid/kg (Maxinutri, Brazil). Body weight, food intake, naso-anal length (NAL), Lee index, retroperitoneal, and mesenteric and epididymal adipose tissues were determined (14).

### Blood biochemical measurements

At 90 days of age, after 12 h of fasting, all rats were euthanized by decapitation and total blood was collected in heparinized tubes and plasma was obtained by centrifugation. Plasma concentrations of glucose, triacylglycerol, insulin, interleukin-6 (IL-6), and IL-10 were measured by kits following the instructions of the manufacturer.

### Adipose tissue harvesting

Fat tissues from mesenteric (MES), epididymal (EPI), and retroperitoneal (RT) tissues were rapidly removed, and their weight was measured on a digital scale (Denver Instruments Company AA-200, USA).

### IL-6 and IL-10 production from adipose tissue incubation

Mesenteric and retroperitoneal fat tissues were cut with scissors into small fragments and incubated at 37°C for 2, 4, and 6 h in Krebs-Ringer bicarbonate buffer containing 5.6 mM glucose and 1.5% BSA, pH 7.4. At the end of the incubation, the media were frozen for further measurement of IL-6 and IL-10.

### Morphological analysis

Samples of approximately 4 mg of MES adipose tissue were collected and fixed in metacarn solution (70% methanol, 20% chloroform, and 10% glacial acetic acid; Sigma-Aldrich, USA) for 24 h. Then, they were stored in 70% alcohol for later histological processing. Stored tissue samples were subjected to dehydration in an alcoholic battery, clearing in n-butyl alcohol, and inclusion in histological paraffin. Histological slides with 7-μm-thick sections were then prepared and stained with hematoxylin and eosin (HE) and analyzed by light microscopy (Carl Zeiss^TM^ Primo Star, Germany), observing the standard morphological characteristics of the tissues. For the morphometric analyses of the adipose tissue, the slides were photomicrographed, obtaining 20 fields at 400×magnification, and the cross-sectional area of 75 adipocytes per animal was measured and the density of adipocytes per mm^2^ (15) was determined (Image Pro Plus 6.0 software, USA).

### Macrophage isolation and functional parameter measurements

Ten mL of sterile phosphate-buffered saline (PBS) were injected into the peritoneum and the resident macrophages were harvested and centrifuged (290 *g*, 4°C for 5 min), washed, and further resuspended in RPMI 1640 medium. Then, peritoneal cells were incubated in tissue culture plates for 1 h and washed 3 times with PBS to remove non-adherent cells. Macrophage enrichment was assayed by May-Grünwald and Giemsa stains and light microscopy, and 95% of the cells in the visual field were identified as macrophages.

### Adhesion assay

The adhesion assay was performed according to Rosen et al. (16). Briefly, peritoneal macrophages obtained as described above were briefly suspended in an RPMI culture medium and plated at a density of 10^6^ cells/well onto flat-bottomed 96-well plates. After incubation for 1 h at 37°C, plates were washed 3 times with PBS, and the adherent cells were fixed with methanol. Cells were stained with Giemsa solution (10%) for 10 min before being rinsed with water, and the retained dye was solubilized in methanol. Staining was quantified by measuring absorbance at 460 nm in an automatic plate reader, and adhesion is reported as absorbance (A_460_) ×10^5^ cells.

### Phagocytosis

All macrophage functional parameters were determined as described by Bonatto et al. (17). In a set of 96-well flat-bottomed tissue culture plates, peritoneal macrophage suspensions were added (0.1 mL containing 10^5^ cells) and incubated for 1 h at 37°C. Then, 10 μL of neutral-red-stained zymosan (10^8^ particles/mL) was added to each well and incubated for 30 min. The cells were then fixed with Baker formal-calcium solution (4% formaldehyde, 2% sodium chloride, 1% calcium acetate) for 30 min, washed 2 times, and centrifuged at 453 *g* for 5 min at 4°C. The neutral-red stain was solubilized by adding 0.1 mL of acidified alcohol (10% acetic acid, 40% ethanol in distilled water) and after 30 min, the absorbance at 550 nm of each well was read on a plate reader. Phagocytosis was calculated from a standard curve constructed from known amounts of stained zymosan and results are reported as zymosan ×10^7^ cells/adhesion.

### Lysosomal volume

To assess the total content of the cationic vesicles of peritoneal macrophages, the uptake of the neutral-red cationic dye was measured. Twenty μL of neutral-red (3%) in PBS was added to 0.1 mL of cell suspension (10^6^ cells/mL) per well and incubated for 1 h. Then, the cells were washed twice with PBS after centrifugation (453 *g* for 5 min). Neutral red was solubilized by a 30-min incubation with 0.1 mL of 10% acetic acid plus 40% ethanol solution. The absorbance was read at 550 nm and the results are reported as Abs/adhesion.

### Superoxide anion production

Peritoneal macrophages (10^5^ cells in 0.1 mL) were incubated for 1 h in PBS at 37°C in the presence of reduced nitroblue tetrazolium yielding blue formazan. By adding 0.45 mL of acetic acid, the reaction was stopped, and the absorbance was read at 560 nm with the results reported as Abs/adhesion.

### Peroxide production

Peritoneal macrophages (10^5^ cells in 0.1 mL) were incubated for 1 h with glucose (5 mM), phenol red solution (0.56 mM), and horseradish peroxidase (8.5 U/mL) in the dark at 20°C. The concentration of hydrogen peroxide (H_2_O_2_) was determined from a standard curve prepared in parallel. The absorbance was read at 620 nm on a plate reader, and the results are reported as μmol/adhesion.

### Statistical analyses

Statistical tests were performed using GraphPad Prism version 8.0 for Windows (GraphPad Software, USA). Data are reported as means±SD, being evaluated for normality (Shapiro-Wilk test) and homoscedasticity (Bartlett test). The Student's *t*-test was used to test for differences between control and small litter groups (C *vs* SL). Ordinary two-way ANOVA was used to analyze fat tissue weight and blood biochemical parameters (factors: litter size and supplementation). The two-way ANOVA with repeated measures test was used for body weight (factors: litter size × supplementation × time), followed by Tukey's *post hoc* test. P<0.05 was used to indicate statistical significance.

## Results

At 30 and 60 days, both the C and SL groups increased the percentages of body weight and food intake (P<0.05) compared to after weaning (21 days) (Supplementary Table S1), where the values for the SL group were significantly higher in both parameters. The SL group showed higher Lee index values (Supplementary Table S1) and were smaller as measured by NAL compared to C (P<0.05). RT, MES, and EPI adipose masses increased by 290, 135, and 294%, the adiposity index was 240% higher in the SL group compared to C (P<0.05). Plasma concentration of glucose was normal despite the hypoinsulinemia, and plasma concentrations of pro- and anti-inflammatory IL-6 and IL-10 were, respectively, higher in the SL group compared to C (P<0.05). Therefore, we found that 60-day-old SL rats were obese, hyperphagic, normoglycemic, hypoinsulinemic, and had elevated concentrations of IL-6 and IL-10. All these findings demonstrated that, at this age, an initial metabolic disturbance was established and the 30-day FO supplementation was started.

As can be seen in Supplementary Table S2, the percent body weight gain in Ob and ObFO groups was similar (P>0.05 ObFO *vs* Ob) from days 60 to 90 and remained higher compared to the lean groups (P<0.05 *vs* C and FO). Percent food intake in ObFO rats was reduced compared to non-supplemented obese rats (P<0.05; ObFO *vs* Ob). Lee index and percent body weight gain in FO supplemented rats was lower but not significantly different compared to obese rats (P>0.05; ObFO *vs* Ob). RT, EPI, and MES adipose tissue gain after 60 days of life (Supplementary Table S1) in the obese group (Ob) increased by 199, 169, and 208%, respectively, compared to the C group (P<0.05). Adipose tissue mass of the FO group increased by 9.6% (RT), 7.7% (EPI), and 11% (MES), respectively, compared to the C group (P<0.05). FO supplementation to the obese group (ObFO) induced lower adipose mass gain, with RT, EPI, and MES 15.7%, 16.5%, and 29.6%, respectively, lower compared to the obese group (P<0.05; ObFO *vs* Ob). However, it was significantly higher compared to the FO group (P<0.05 ObFO *vs* FO). These findings from adipose mass weight were corroborated by the adiposity index.

Plasma insulin concentration (Supplementary Table S2) remained lower on day 90 in the obese group (P<0.05; Ob *vs* C). FO given to the obese group (ObFO) reestablished insulin concentration to normal values (P>0.05 *vs* C and FO). Blood glucose levels were not altered (Supplementary Table S2) by FO supplementation in the lean groups (P>0.05; FO *vs* C). Obesity caused hyperglycemia (P<0.05; Ob *vs* C), and FO supplementation to obese rats (ObFO) prevented hyperglycemia (P<0.05; ObFO vs Ob) with similar results to lean rats (P>0.05; ObFO *vs* C and FO). Triacylglycerolemia (Supplementary Table S2) was significantly reduced by fish oil in both supplemented groups (FO and ObFO) compared to the non-supplemented groups (P<0.05; C and Ob). Furthermore, no difference was found between control and obese groups (P>0.05; Ob *vs* C). IL-6 and IL-10 plasma concentrations were not different between the four groups (P>0.05).

**Figure 1:**
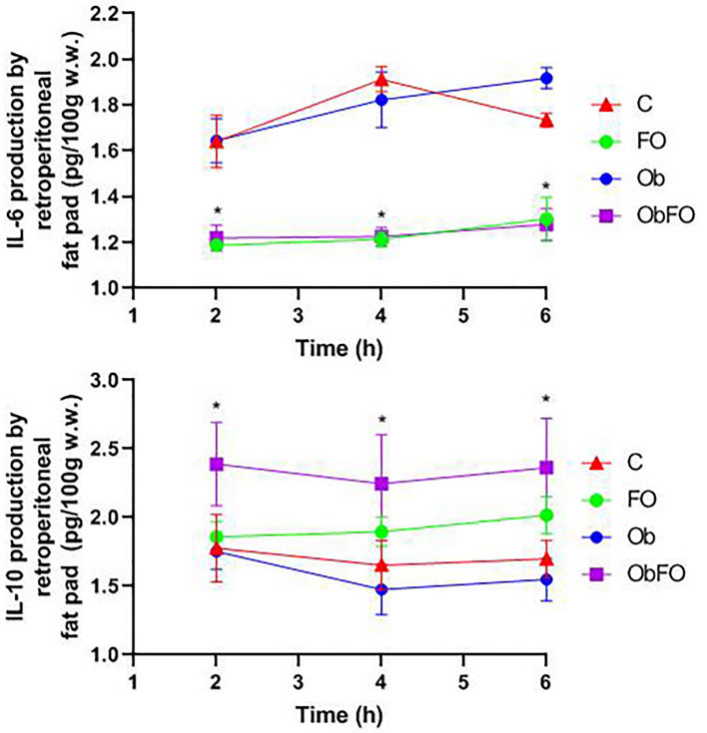
Interleukin (IL)-6 and IL-10 production by retroperitoneal adipose tissue in the control (C), fish oil supplemented (FO), obese (Ob), and obese fish oil supplemented (ObFO) rats after incubation for 2, 4, and 6 h. Data are reported as means±SD *P<0.05 compared to non-supplemented group (two-way ANOVA). w.w., wet weight.

**Figure 2:**
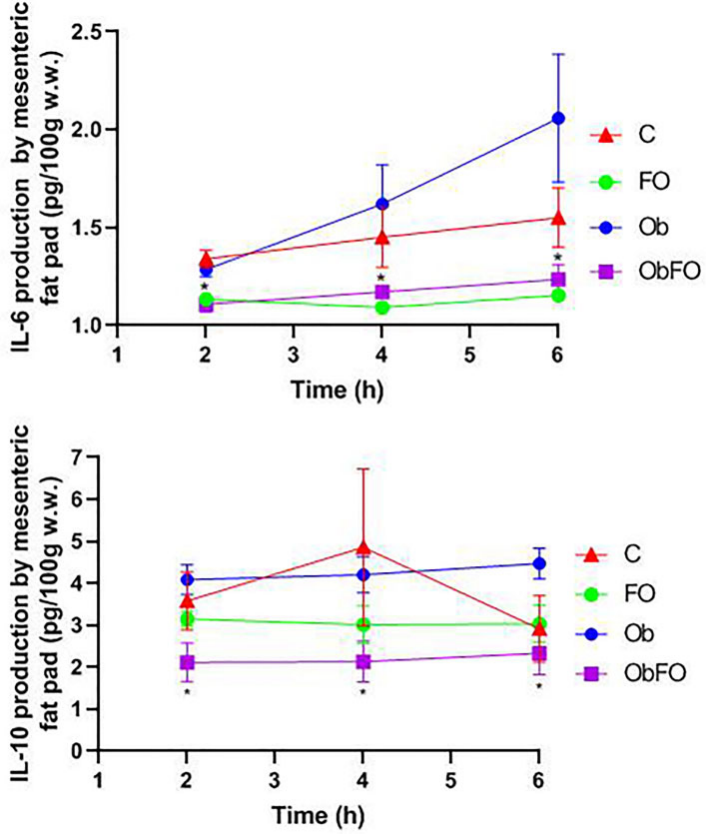
Interleukin (IL)-6 and IL-10 production by mesenteric adipose tissue obtained from control (C), fish oil supplemented (FO), obese (Ob), and obese fish oil supplemented (ObFO) rats after incubation for 2, 4, and 6 h. Data are reported as means±SD. *P<0.05 compared to non-supplemented group (two-way ANOVA). w.w., wet weight.

The *ex vivo* production of IL-6 and IL-10 from RT and MES fat tissues incubated for 2, 4, and 6 h from the 4 groups is shown in Figures 1 and 2. IL-6 production by RT and MES fat pads from FO supplemented groups (FO and ObFO) was significantly lower compared to the non-supplemented groups (P<0.05 *vs* C and Ob), which was maintained at 4 and 6 h. In the obese groups, IL-6 production at 2 and 4 h was significantly higher compared to FO supplemented groups, being higher at the end of 6 h of incubation in all adipose tissues compared to the control group (P<0.05 *vs* C). IL-10 production by MES fat tissue from the supplemented groups (FO and ObFO) was significantly lower compared to the non-supplemented obese group (P<0.05 *vs* Ob) and did not change at any incubation time point. In the control group (C), IL-10 production was lower compared to the obese group (P<0.05; C *vs* Ob) only at 6 h of incubation. On the other hand, IL-10 production by RT fat tissue at 2 h of incubation was significantly higher in the obese FO supplemented compared to all other groups (P<0.05). At 4 and 6 h of incubation, IL-10 production was not different in the FO supplemented group, but it was markedly higher compared to obese non-supplemented group (P<0.05; ObFO *vs* Ob). Obese rats showed the lowest IL-10 production.

**Figure 3:**
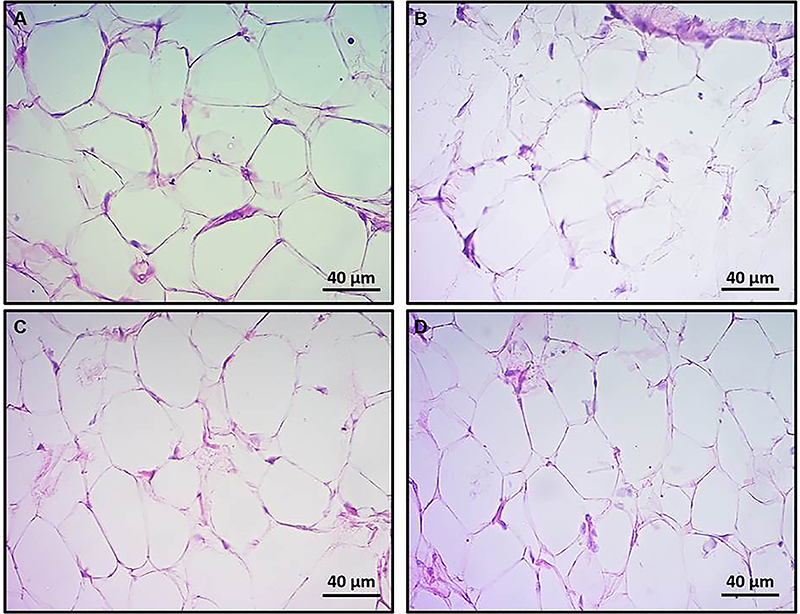
Representative photomicrographs of mesenteric adipose tissue from control (**A**), fish oil supplemented (**B**), obese (**C**), and obese fish oil supplemented (**D**) groups. Seven-µm-thick cryosections stained with hematoxylin-eosin were used for measurement of cross-sectional area and cell density. Scale bar 40 µm.

**Figure 4:**
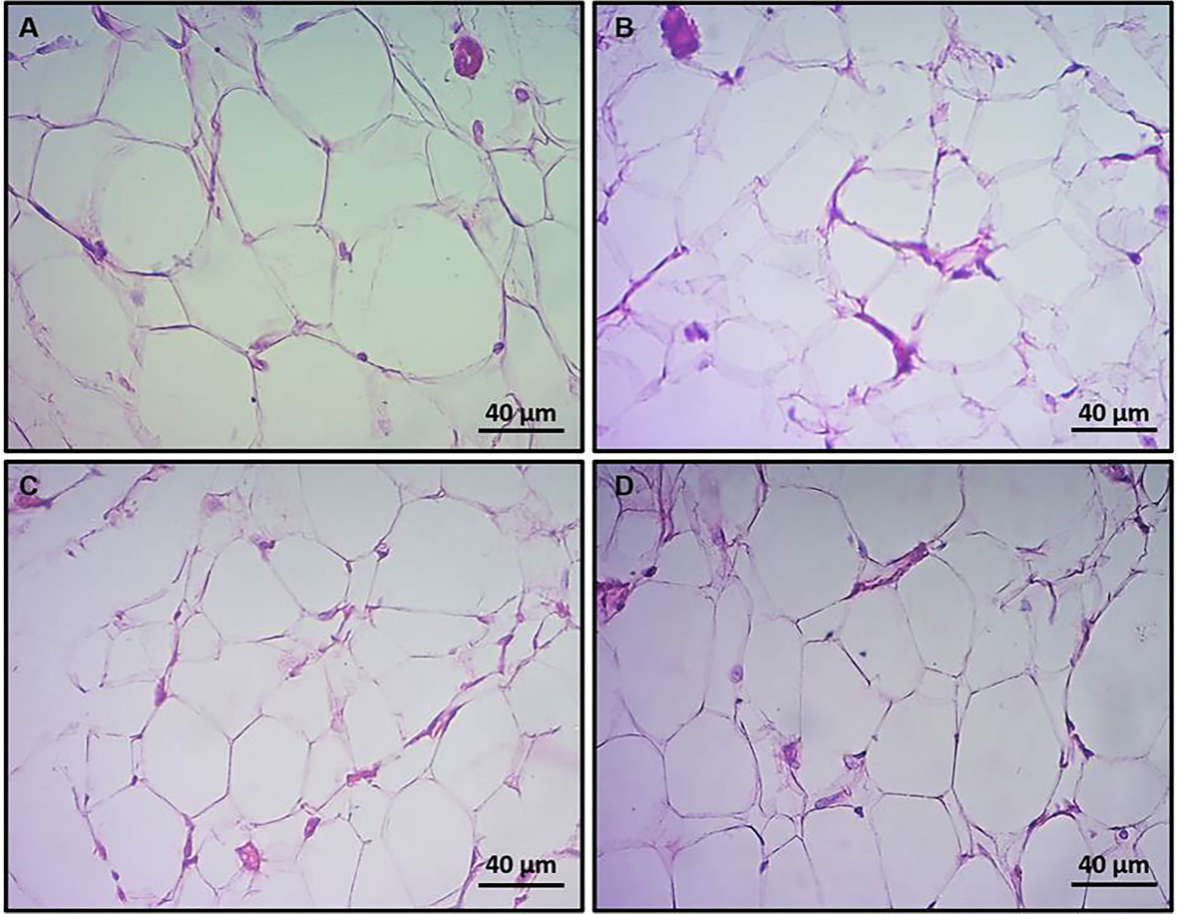
Representative photomicrographs of retroperitoneal adipose tissue from control (**A**), fish oil supplemented (**B**), obese (**C**), and obese fish oil supplemented (**D**) groups. Seven-µm-thick cryosections stained with hematoxylin-eosin were used for measurement of cross-sectional area and cell density. Scale bar 40 µm.

**Figure 5:**
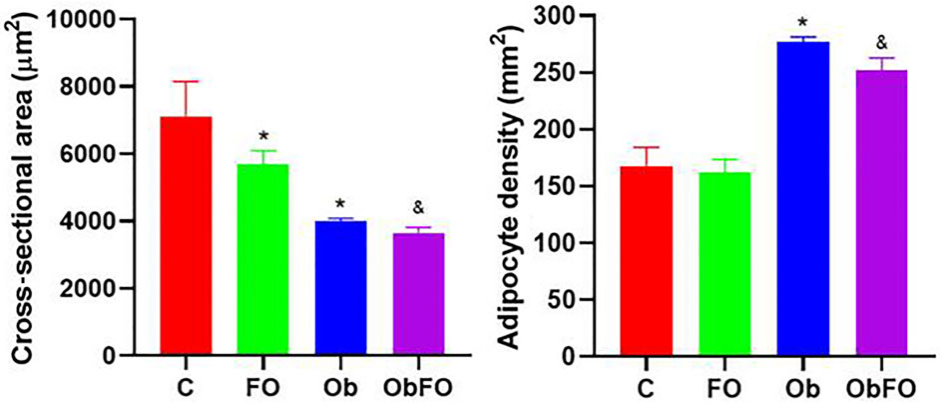
Cross-sectional area and density of adipocytes of the mesenteric adipose tissue from control (C), fish oil supplemented (FO), obese (Ob), and obese fish oil supplemented (ObFO) groups. Data are reported as means±SD. *P<0.05 compared to C; ^&^P<0.05 compared to Ob (ANOVA).

Representative photomicrographs of MES and RT adipose tissues are shown in Figures 3 and 4, respectively. Morphometry analysis of MES adipose tissue showed a significant increase in adipocyte density (mm^2^) in Ob (Figure 5) compared to C (P<0.05). ObFO showed a slight reduction in adipocyte density, but no difference was observed compared to the Ob group (P>0.05), although these levels were higher when compared to FO (P<0.05). The cross-sectional area was markedly lower in obese rats compared to control (P<0.05), and FO supplementation did not have an effect (P>0.05; ObFO *vs* Ob). FO rats also showed reduction in the cross-sectional area compared to the control group, but higher than the ObFO group (P<0.05).

**Figure 6:**
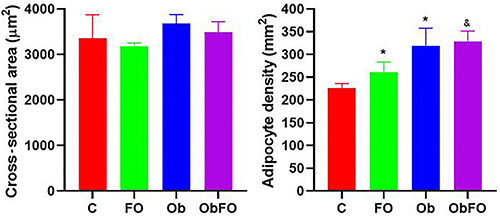
Cross-sectional area and density of adipocytes of the retroperitoneal adipose tissue from control (C), fish oil supplemented (FO), obese (Ob) and obese fish oil supplemented (ObFO) groups. Data are reported as means±SD. *P<0.05 compared to C; ^&^P<0.05 compared to Ob (ANOVA).

RT adipose tissue morphometry behaved differently compared to MES adipose tissue (Figure 6). The adipocyte cross-sectional area of RT white adipose tissue was not different between groups (P>0.05). On the other hand, adipocyte density of FO rats was 15% higher compared to control (P<0.05). In obese rats, the adipocyte density was 53% higher compared to control (P<0.05). FO supplementation to obese rats did not cause any effect on adipocyte density (P>0.05 ObFO *vs* Ob), but it did increase adipocyte density by 26% compared to FO supplemented lean rats (P<0.05 ObFO *vs* FO).

Functional parameters from peritoneal macrophages of control (C) and small litter (SL) rats at 60 days are shown on Table 1. The number of adherent cells in the SL group was 1.7-fold higher compared to C (P<0.05). Phagocytosis and lysosomal volume in SL rats were 2.2- and 3.3-fold lower compared to C (P<0.05). Anion superoxide and H_2_O_2_ production in SL rats was 7.2- and 3-fold lower compared to C (P<0.05), respectively.

**Table 1:** Adhesion, phagocytosis, lysosomal volume, anion superoxide, and hydrogen peroxide of peritoneal macrophages from control (C) and small litter (SL) rats at 60 days old.

Groups	Adherent cells (A_460_)×10^5^ cells	Phagocytosis (zymosan×10^7^/adherent cells)	Lysosomal volume (Abs/adherent cells)	Anion superoxide (Abs/adherent cells)	Hydrogen peroxide (μmol/adherent cells)
C	0.068±0.004	7.194±0.418	1.757±0.0501	8.287±0.821	9.114±0.575
SL	0.119±0.021*	3.266±0.124*	0.526±0.0106*	1.135±0.081*	3.086±0.359*

Data are reported as means±SD (n=10 rats/group). *P<0.05 *vs* Control; *t*-test.

Functional parameters from the 4 groups at 90 days are shown in Table 2. The number of adherent cells in obese rats increased 3.9-fold compared to C (P<0.05; Ob *vs* C). Thirty days of fish oil supplementation to obese rats (ObFO) reduced adhesion 1.9- and 5.7-fold compared Ob (P<0.05) and FO (P<0.05). Fish oil supplementation to lean rats reduced macrophage adhesion by 2.9-fold compared to C group (P<0.05). Phagocytic capacity of the FO group was increased 2.6-fold compared to control (P<0.05). Obesity markedly reduced by 4.3-fold phagocytic capacity of peritoneal macrophages compared to control (P<0.05). ObFO increased phagocytosis by 1.5-fold compared to Ob (P<0.05), and it was 7.6-fold lower compared to FO (P<0.05). FO increased lysosomal volume 1.3-fold compared to control (P<0.05). Obesity reduced lysosomal volume by 1.2-fold compared to C group (P<0.05), and FO supplementation to obese rats (ObFO) reduced lysosomal volume 1.2- and 2-fold compared to Ob (P<0.05) and FO (P<0.05), respectively. Anion superoxide and H_2_O_2_ production by peritoneal macrophages from FO was 1.6- and 2.8-fold higher compared to C group (P<0.05). Obesity reduced anion superoxide and H_2_O_2_ production in Ob by 2.5- and 3.9-fold compared to C group (P<0.05). ObFO increased anion superoxide and H_2_O_2_ production by 2.1- and 1.8-fold compared to Ob (P<0.05). Anion superoxide and H_2_O_2_ production of the FO group was 1.9- and 5.9-fold higher compared to ObFO (P<0.05).

**Table 2:** Adhesion, phagocytosis, lysosomal volume, anion superoxide, and hydrogen peroxide of peritoneal macrophages from control, fish oil supplemented (FO), obese (Ob), and obese fish oil supplemented (ObFO) rats at 90 days old.

Groups	Adherent cells (A_460_)×10^5^ cells	Phagocytosis (zymosan×10^7^/adherent cells)	Lysosomal volume (Abs/adherent cells)	Anion superoxide (Abs/adherent cells)	Hydrogen peroxide (μmol/adherent cells)
C	0.085±0.005	21.729±1.736	0.651±0.032	7.905±0.554	16.336±0.897
FO	0.029±0.002*	56.677±5.714*	0.868±0.136*	12.954±0.849*	45.573±3.862*
Ob	0.328±0.005*	5.027±0.662*	0.543±0.016*	3.122±0.201*	4.123±0.136*
ObFO	0.167±0.013&,#	7.521±0.555&,#	0.469±0.044&,#	6.578±0.462&,#	7.713±0.277&,#

Data are reported as mean±SD (n=10 rats/group). *P<0.05 *vs* Control; ^&^P<0.05 vs FO; ^#^P<0.05 *vs* Ob (ANOVA).

## Discussion

In both industrialized and developing countries, obesity has been related to MS, where individuals with MS have a higher risk of developing cardiovascular diseases and type 2 diabetes (1,18). These metabolic disorders are, in part, linked to adipose tissue secretions, collectively known as adipokines (19), and low-level chronic inflammation. Our results showed that at 60 days of age rats from the SL group were obese, normoglycemic, hypoinsulinemic, and presented higher concentrations of IL-6 and IL-10, pro- and anti-inflammatory cytokines, respectively, which can be collectively translated as metabolic disturbance.

By the age of 90 days, all rats were hyperphagic, obese (higher fat depots), hyperglycemic, hypoinsulinemic, hypertriacylglicerolemic, and *ex vivo* IL-6 secretion by incubated fat tissues was higher. Therefore, MS features were strong at this stage. The main dietary intervention to treat MS is caloric restriction aiming at weight loss. We chose a different intervention and investigated the effect of FO supplementation on MS in obese rats. Docosahexaenoic (DHA) and eicosapentaenoic (EPA) n-3 fatty acids, found in high amounts in fish oil, have been reported to reduce fat mass from RT and EPI tissues, and such effect was partially mediated by the thermogenic activity of brown adipose tissue (20). Our findings corroborated these results showing a fat mass reduction in RT and EPI tissues by FO, and we expanded these findings to the mesenteric (MES) fat depot. Therefore, the distribution of adipose tissue was affected by the n-3 PUFA present in fish oil.

Increase of body weight, fat mass, and plasma triacylglycerol concentration are strongly correlated with MS. Overweight and obese individuals with MS ingesting 0.3-3.0 g n-3 PUFA/day showed reduction of body weight and body fat (21), and our results demonstrated that FO supplementation lowered body weight and TAG concentration, which means that DHA and EPA act as natural hypolipidemics (22), possibly preventing the progress of MS (23). Even in a non-obese condition (FO group), fish oil introduction reduced plasma TAG concentration. On the other hand, some studies did not find any significant reduction in body weight or in some fat depots in n-3 PUFA supplemented groups (9).

Accumulation of visceral adipose tissue is an obesity-related metabolic disorder factor associated with MS (24), insulin resistance, and dyslipidemia (25). It has been reported that increased adiposity contributes to dyslipidemia, glucose intolerance, and insulin resistance leading to cardiovascular disease. Indeed, our obese rats developed hypertriacylgliceromia, hyperglycemia, and hypoinsulinemia. These results suggested that hyperglycemia might be caused by low insulinemia, but we cannot rule out insulin resistance development. Fish oil supplementation returned these blood biochemical parameters to normal concentrations. It has been shown that n-3 PUFA reduces lipid uptake by adipocytes via suppressing lipoprotein lipase (26), while increasing lipolysis via beta-adrenergic receptors (27) and expression of the mitochondrial oxidative apparatus leading to beta-oxidation of fat in white adipose tissue (28). Therefore, a comprehensive understanding of the metabolism of carbohydrate and fat in visceral adipose tissue and plasma will help to better understand the effect of n-3 PUFA on lipotoxicity in non-adipose organs.

Adipose tissue has high plasticity and can show alterations in the number of fat cells, the weight of several fat stores, the size of adipocytes, and the number of cell types contained in the tissue (29). Accumulation of fat in white adipose tissues (WAT) causes obesity by hyperplasia or hypertrophy (30). Mesenteric adipose tissue from obese rats had adipocytes of higher density and lower cross-sectional area, meaning hyperplasia and low volume. Retroperitoneal WAT adipocytes, in turn, had no changes in the cross-sectional area, but a higher adipocyte density in the obese groups, either supplemented or not. FO supplementation was not able to reduce hyperplasia in these tissues, but it reduced the cross-sectional area of the mesenteric adipose tissue.

As adipocytes grow larger, they become insulin-resistant, hyperlipolytic, and an active endocrine “organ”. The hyperplasia of the two visceral adipose tissues in the obese rats will eventually increase the amount of TAG depot, and lead to larger adipocyte volume, which will later secrete adipokines associated with MS.

Another hallmark found in MS patients is the inflammatory marker IL-6 in subcutaneous adipose tissue and plasma (31). *Ex vivo* IL-6 production by mesenteric and retroperitoneal fat mass was increased after 6 h of incubation. Fish oil supplementation caused a marked reduction on IL-6 production by both adipose tissues, in both lean and obese rats. It seems that the secretory pattern is altered by changes in adiposity, i.e. higher adiposity means higher IL-6 production and lower adiposity reflects lower IL-6 secretion. In plasma, there was a tendency of higher IL-6 but that was not significant. Regarding anti-inflammatory cytokine IL-10, incubated retroperitoneal fat tissue had a lower secretion compared to obese fish oil supplemented animals, but in the mesenteric fat tissue, we observed the opposite. These findings indicated that the profile of cytokines secretion is different among different WAT, in agreement with Belzung et al. (32), who demonstrated that epididymal and retroperitoneal adipose tissues are more responsive to n-3 PUFA then mesenteric adipose tissue.

Ibrahim (33) describes the structure of WAT as having a large number of adipocytes and non-adipocyte cellular components, including macrophages. Innate immunity plays a key role in inflammation and pathogen destruction. The inflammatory response begins with alterations in the vessel caliber, favoring the exit of plasma and leukocytes from blood, leading to binding and transmigration of blood white cells to the tissue (34,35). Obesity is an on-going inflammatory process of lower intensity compared to classical inflammatory diseases.

Macrophage adhesion depends on several receptors for binding to endothelial cells and migration into tissues. Then, macrophage phagocytic activity takes place, consisting of a major membrane-associated event in which particles bound to specific or non-specific membrane receptors are internalized, fused with lysosomes, and then digested (36). The data of this study showed that obese rats have a weaker innate immunity compared to lean rats.

Mahoney et al. (37) reported that under normal conditions, the macrophage plasma membrane turnover occurs in about 33 min. Therefore, turnover of phospholipids from the macrophage plasma membrane might occur rapidly. Several studies report that different types of fatty acids modify the immune system (38,39). Monocytes/macrophages are not the main “target” of fish oil therapy, but when dietary n-3 PUFA changes the plasma profile, it might simultaneously modify the lipid content and the composition of these cells, altering membrane structure and cellular function. Indeed, our data corroborated this information. Compared to the non-supplemented obese rats, fish oil supplemented obese rats showed improvement in all innate immunity functions of peritoneal macrophages, including the respiratory burst in the generation of reactive O_2_ metabolites, which are toxic for pathogens and tumor cells. Increased phagocytosis induced by fish oil supplementation results from altered membrane fluidity (40). This effect, associated with decreased adhesiveness, is important in the therapy of inflammatory and autoimmune disturbances.

In summary, our results suggested that as soon as MS signs are identified, fish oil supplementation can alleviate the morphological and biochemical effects of the syndrome and also improve the innate immune response. Finally, the approach should be tested in human subjects to validate fish oil dosage and timing for prevention and/or treatment of MS.

## Supplementary Material

Click to view [pdf].
