# Supplementary material for: Fish oil supplementation in obese rats ameliorates metabolic syndrome response
Source: Braz J Med Biol Res. 2024 May 20;57:e13172. doi: 10.1590/1414-431X2024e13172 (PMC11136482; doi:10.1590/1414-431X2024e13172)
Supplement: Supplementary file 1 [file 1414-431X-bjmbr-57-e13172-suppl.pdf]

Table S1. Lee index, naso-anal length, retroperitoneal (RT), mesenteric (MES), epididymal (EPI) mass, adiposity index, plasma concentration of glucose, insulin, interleukin-6 (IL-6), interleukin-10 (IL-10), and other measurements from control and small litter rats at 60 days old.

Group	Lee Index ($\sqrt[3]{\mathbf{p}}$ (g)/NAL (cm)	Naso-anal length (cm)	Body weight gain (% 21–30 and 21–60 days)	Food intake gain (% 21–30 and 21–60 days)	RT (g/100 g b.w.)	MES (g/100 g b.w.)	EPI (g/100 g b.w.)	Adiposity Index (AU)	Plasma glucose (mg/dL)	Plasma insulin (pg/mL)	IL-6 (pg/mL)	IL-10 (pg/mL)
Control	519.89 ± 7.95	21.85 ± 0.43	208.59 ± 9.68 (30 days) 272.88 ± 14.90 (60 days)	218.67 ± 12.46 327.99 ± 25.27	0.42 ± 0.02	0.45 ± 0.01	0.41 ± 0.01	1.28 ± 0.02	105.67 ± 2.06	3.33 ± 0.19	140.90 ± 0.82	318.22 ± 15.94
Small litter	572.13 ± 9.97*	20.35 ± 0.45*	424.22 ± 19.60* (30 days) 520.32 ± 16.43* (60 days)	255.67 ± 14.26* 404.50 ± 32.25*	1.24 ± 0.05*	0.61 ± 0.03*	1.19 ± 0.03*	3.04 ± 0.06*	104.33 ± 1.67	2.74 ± 0.05*	150.54 ± 0.54*	423.99 ± 7.56*

Data are reported as means±SD (n=10 rats/group). *P<0.05 *vs* Control (*t*-test). NAL: naso-anal length; b.w.: body weight; AU: arbitrary units.

Table S2. Lee index, naso-anal length, retroperitoneal (RT), mesenteric (MES), epididymal (EPI) mass, adiposity index, plasma concentration of interleukin-6 (IL-6), interleukin-10 (IL-10), and other measurements from control, fish oil supplemented (FO), obese (Ob), and obese fish oil supplemented (ObFO) rats at 90 days old.

Group	Lee Index ($\sqrt[3]{\mathbf{p}}$ (g)/NAL (cm)	Naso-anal length (cm)	Body weight gain (% 21–90 days)	Food intake gain (% 21–90 days)	RT (g/100 g b.w.)	MES (g/100 g b.w.)	EPI (g/100 g b.w.)	Adiposity Index (AU)	IL-6 (pg/mL)	IL-10 (pg/mL)	Plasma glucose (mg/dL)	Plasma insulin (mg/dL)	Plasma triacylglycerol (mg/dL)
C	519.89 ± 7.95	21.85 ± 0.43	548.50 ± 11.12	445.66 ± 38.15	1.02 ± 0.10	0.90 ± 0.10	1.09 ± 0.07	3.02 ± 0.24	146.34 ± 4.42	309.20 ± 7.20	118.56 ± 5.02	2.78 ± 0.09	74.28 ± 5.41
FO	507.70 ± 20.88	21.25 ± 0.26	524.01 ± 10.12	438.26 ± 35.15	1.12 ± 0.07	0.99 ± 0.11	1.19 ± 0.03*	3.30 ± 0.07*	176.81 ± 24.72	311.24 ± 6.29	116.88 ± 15.28	2.77 ± 0.06	55.01 ± 0.88*
Ob	572.16 ± 9.96*	20.35 ± 0.45*	645.95 ± 22.12*	550.63 ± 41.03*	2.03 ± 0.15*	1.84 ± 0.08*	1.85 ± 0.08*	5.73 ± 0.181*	165.42 ± 15.15	304.26 ± 5.72	159.58 ± 6.32*	2.58 ± 0.02*	91.42 ± 6.13*
ObFO	559.03 ± 16.02[&]	21.10 ± 0.71	616.88 ± 13.34[&]	480.27 ± 32.26[#]	1.71 ± 0.06[&#]	1.29 ± 0.09[&#]	1.55 ± 0.08[&#]	4.56 ± 0.16[&#]	172.10 ± 17.40	288.63 ± 8.45	130.05 ± 7.45[#]	2.76 ± 0.07	62.50 ± 3.50[#]

Data are reported as means±SD (n=10 rats/group). *P<0.05 *vs* Control; [&]P<0.05 vs FO; [#]P<0.05 *vs* Ob (ANOVA). b.w.: body weight; AU: arbitrary units.
